# Perceptions and Misconceptions About Eye Disease Treatment: A Cross-Sectional Study From Jazan, Saudi Arabia

**DOI:** 10.7759/cureus.71218

**Published:** 2024-10-10

**Authors:** Essam A Alhazmi, Osama A Mobarki, Mohammed E Mojiri, Waad K Najmi, Raum A Ayoub, Hani A Al-Ghamdi, Abdulrahman S Jathmi, Kamlah I Samkari, Yazan A Bajawi, Arwa H Alammari, Shahad A Alhazmi, Lujain B Suhaqi, Atyaf A Madkhali, Lujain H Rajab, Abdulaziz A Alagsam

**Affiliations:** 1 Department of Ophthalmology, Prince Mohammed Bin Nasser Hospital, Jazan, SAU; 2 College of Medicine, Jazan University, Jazan, SAU; 3 College of Medicine, Al-Baha University, Al-Baha, SAU; 4 Faculty of Medicine, King Abdulaziz University, Rabigh, SAU

**Keywords:** awareness, cataracts, diabetic patients, eye diseases, glaucoma, jazan, perceptions, public health, saudi arabia, vision impairment

## Abstract

Background: Eye diseases such as cataracts and glaucoma significantly contribute to vision impairment and blindness worldwide. While cataracts are the leading cause of preventable blindness, glaucoma, often referred to as the "silent thief of sight", can lead to irreversible vision loss if untreated. Despite their prevalence, awareness and understanding of these conditions remain low, particularly in regions like Saudi Arabia, where demographic changes and rising diabetes rates exacerbate the burden of eye diseases. This study aims to assess the knowledge and perceptions of cataracts and glaucoma among residents of Jazan to identify gaps and inform public health interventions.

Methods: A cross-sectional online survey was conducted among participants aged 18 years and older in Jazan. The structured questionnaire assessed demographic information, awareness of cataracts and glaucoma, understanding of symptoms, and perceptions of treatment options. Data collection took place over four weeks, with statistical analysis performed using descriptive statistics to summarize findings.

Results: A total of 426 participants completed the survey. Of these, 43.9% (n = 186) were aware of cataracts, while only 28.1% (n = 119) recognized glaucoma. Awareness of cataract symptoms varied, with 43.4% (n = 184) identifying blurred vision; however, 71.5% (n = 303) reported being unaware of glaucoma. A significant majority (68.2%, n = 289) recognized surgical treatment for cataracts, but only 27.8% (n = 118) were aware of treatment options for glaucoma. High levels of uncertainty about both conditions suggest a critical lack of understanding in the community.

Conclusion: This study emphasizes the need for improved awareness and understanding of cataracts and glaucoma in Jazan, Saudi Arabia. By implementing targeted public health strategies, healthcare providers can enhance knowledge, encourage regular eye examinations, and ultimately reduce the burden of visual impairment in the community.

## Introduction

Eye diseases, particularly cataracts and glaucoma, are significant public health concerns worldwide, contributing substantially to vision impairment and blindness. Cataracts, characterized by the clouding of the eye's lens, affect millions of individuals globally, with aging being the primary risk factor [1,2]. They can lead to blurred vision, difficulty in night driving, and eventual vision loss if left untreated. According to the World Health Organization (WHO), cataracts are responsible for approximately 51% of world blindness, making early detection and timely intervention crucial for preserving vision [3-7].

Glaucoma, on the other hand, is often referred to as the "silent thief of sight" due to its insidious onset and gradual progression. It is primarily characterized by increased intraocular pressure, which can damage the optic nerve over time. If untreated, glaucoma can lead to irreversible vision loss, affecting millions worldwide [6,8]. The prevalence of glaucoma increases with age, and it is estimated that by 2020, nearly 80 million people globally will be affected by the disease [5-8]. Early detection through regular eye examinations is vital for managing glaucoma effectively [7,8].

In Saudi Arabia, the burden of eye diseases is particularly pronounced due to demographic factors, including an aging population and increasing rates of diabetes, which is associated with diabetic retinopathy, another leading cause of vision impairment [1-3]. Despite advancements in medical care, awareness and understanding of these conditions among the general population remain low. Studies have indicated that misconceptions about symptoms, treatment options, and the importance of regular eye exams contribute to delayed diagnosis and treatment.

The current study aims to assess the knowledge and perceptions of cataracts and glaucoma among residents of Jazan, Saudi Arabia, to identify gaps in understanding and inform future public health interventions. By evaluating the level of awareness and common misconceptions, this research seeks to highlight the need for targeted educational efforts to improve eye health outcomes in the community.

## Materials and methods

Study design

This study employed a cross-sectional design to investigate the knowledge and perceptions of eye diseases, specifically cataracts and glaucoma, among residents of Jazan, Saudi Arabia. An online survey was utilized to collect data from a diverse participant pool, allowing for flexibility in response time and accessibility.

Study participants

Participants were recruited from the general population of Jazan. Specific inclusion criteria were established to ensure a representative sample. Individuals aged 18 years and older, residing in Jazan, were eligible to participate. The survey link was disseminated through various social media platforms, community groups, and local educational institutions.

Data collection instrument

Data collection was conducted using a structured online questionnaire designed to assess various aspects of participants’ knowledge and perceptions regarding cataracts and glaucoma (Appendix). The questionnaire comprised several sections. The first section collected demographic information, including age, sex, educational level, occupation, and history of eye disease within the family.

The second section focused on participants' awareness of cataracts and glaucoma. Questions included whether participants had heard of these conditions, followed by inquiries about specific symptoms associated with each disease. For example, participants were asked to identify symptoms such as blurred vision, halos around lights, or increased sensitivity to light.

The third section aimed to assess participants' understanding of available treatment options. Respondents were queried about their perceptions of treatment methods, including surgical intervention, medication, and lifestyle changes. Open-ended questions were also included to capture any additional beliefs or misconceptions participants might hold about these diseases and their treatment.

To ensure clarity and reliability, the questionnaire was pre-tested on a small sample before the main data collection. This allowed for adjustments based on participant feedback regarding question clarity and comprehension.

Survey administration

The online survey was administered over a four-week period. To maximize participation, reminders were sent through social media and community forums. Informed consent was obtained from all participants prior to accessing the questionnaire, ensuring that they understood the study's purpose and their rights as respondents. The survey was designed to guarantee anonymity and confidentiality, with no identifying information collected.

Statistical analysis

Statistical analysis involved the use of descriptive statistics to summarize demographic characteristics and survey responses. Frequencies and percentages were calculated for categorical variables, providing a comprehensive overview of participants’ knowledge and perceptions regarding cataracts and glaucoma. Data were analyzed using IBM SPSS Statistics for Windows, Version 26 (Released 2019; IBM Corp., Armonk, New York, United States), with results presented in tables for clarity.

## Results

Demographic characteristics of participants

A total of 426 participants were included in the study, with a gender distribution of 43.9% male (n=186) and 55.7% female (n=236). The majority of participants were aged between 18 and 45 years (89.4%, n=379), while 9.2% (n=39) were in the 46-60 age group, and only 0.9% (n=4) were over 60 years old. In terms of education, 59.7% (n=253) held a bachelor's degree, followed by 30.7% (n=130) with secondary education. A smaller percentage reported other educational backgrounds, including primary education (0.7%, n=3) and intermediate education (0.2%, n=1) (Table 1).

**Table 1:** Demographic characteristics of study participants (N=426) Data presented are based on responses from 426 participants. Values are represented as n (%) for each characteristic.

Characteristic		Count (n)	Percentage (%)
Sex	Male	186	43.9
	Female	236	55.7
Age Group	18-45 years	379	89.4
	46-60 years	39	9.2
	Over 60 years	4	0.9
Level of Education	Bachelor's Degree	253	59.7
	Secondary Education	130	30.7
	Primary Education	3	0.7
	Intermediate Education	1	0.2
	Other (specify)	35	8.3

Knowledge and perceptions regarding cataracts

Participants' awareness of cataracts was measured, revealing that 43.9% (n=186) were aware of the condition, while 55.7% (n=236) reported they were not. Understanding the symptoms of cataracts varied: 43.4% (n=184) identified blurred vision as a symptom, while 19.1% (n=81) associated unclear vision with aging, and 25.2% (n=107) were uncertain about the symptoms. Regarding treatment options, a significant majority (68.2%, n=289) recognized surgical intervention as a treatment for cataracts, while 27.8% (n=118) were uncertain about available treatment options, and only 3.5% (n=15) mentioned medication as a potential treatment (Table 2).

**Table 2:** Knowledge and perceptions regarding cataracts (N=422) Data presented are based on responses from 426 participants. Values are represented as n (%) for each characteristic.

Variable		Count (n)	Percentage (%)
Awareness of Cataracts	Aware of Cataracts (Yes)	186	43.9
	Not Aware of Cataracts (No)	236	55.7
Understanding of Cataracts	Identified Symptoms (Blurred Vision)	184	43.4
	Perception of Aging Impact (Unclear Vision Due to Aging)	81	19.1
Uncertainty Regarding Cataracts	Surgical Intervention Recognized	289	68.2
	Uncertain About Treatment Options	118	27.8
	Awareness of Medication as a Treatment Option	15	3.5

Knowledge of glaucoma and its implications

The study assessed participants' awareness of glaucoma, revealing that only 28.1% (n=119) were aware of the disease, while a substantial 71.5% (n=303) reported being unaware. When evaluating understanding of glaucoma, 26.9% (n=114) recognized that it could cause damage to the optic nerve, and 11.8% (n=50) were aware of elevated intraocular pressure as a related condition. Notably, a significant portion of participants (53.1%, n=225) expressed uncertainty regarding glaucoma, indicating a lack of understanding of its implications (Table 3).

**Table 3:** Knowledge of glaucoma and its implications (N=422) Data presented are based on responses from 426 participants. Values are represented as n (%) for each characteristic.

Variable		Count (n)	Percentage (%)
Awareness of Glaucoma	Aware of Glaucoma (Yes)	119	28.1
	Not Aware of Glaucoma (No)	303	71.5
Understanding of Glaucoma	Recognized Impact on Vision (Damage to Optic Nerve)	114	26.9
	Awareness of Elevated Intraocular Pressure	50	11.8
	Uncertainty Regarding Glaucoma	225	53.1

## Discussion

The findings from this study reveal significant gaps in knowledge and perceptions regarding cataracts and glaucoma among residents of Jazan, Saudi Arabia. Despite the high prevalence of these conditions, awareness levels were found to be low, with only 43.9% of participants recognizing cataracts and 28.1% acknowledging glaucoma. Furthermore, understanding of the symptoms and treatment options for these eye diseases was inadequate, highlighting a critical need for improved educational initiatives.

The low level of awareness regarding cataracts and glaucoma poses serious public health implications [3-7]. Cataracts, being a leading cause of preventable blindness worldwide, require early detection and timely intervention. Similarly, glaucoma is a significant contributor to irreversible vision loss [5-9]. The study’s findings indicate that many individuals may remain undiagnosed or fail to seek treatment, ultimately leading to adverse health outcomes. This underscores the necessity for targeted awareness campaigns that not only inform the public about the existence of these conditions but also educate them on recognizing symptoms and the importance of regular eye examinations.

Participants demonstrated varying degrees of understanding regarding the symptoms associated with cataracts and glaucoma [10-13]. While blurred vision was identified by a notable percentage of participants as a symptom of cataracts, misconceptions surrounding aging-related vision changes may obscure the recognition of cataracts among older adults. The lack of awareness about symptoms associated with glaucoma, such as peripheral vision loss and elevated intraocular pressure, further emphasizes the critical need for community-based educational programs that effectively communicate the early warning signs of these diseases.

The study also revealed that a significant portion of participants had misconceptions about treatment options. While surgical intervention was recognized by a majority as a viable treatment for cataracts, the limited acknowledgment of medication as a treatment for glaucoma highlights a knowledge gap. This lack of understanding may lead to delayed treatment or avoidance of necessary medical care, which can have long-term implications for visual health [11-15]. Public health initiatives should focus on disseminating clear and accurate information about available treatment options and the importance of adherence to prescribed regimens.

Limitations

This study has several limitations. First, as a cross-sectional survey, it captures knowledge and perceptions at a single point in time, which may not reflect changes over time. Second, the reliance on self-reported data could lead to response bias, as participants may have provided socially desirable answers. Additionally, the use of an online survey may have excluded individuals without internet access or those less familiar with technology, potentially affecting the representativeness of the sample. Lastly, the study was conducted in a single region, Jazan, limiting the generalizability of the findings to other areas of Saudi Arabia or internationally.

## Conclusions

This study underscores a critical lack of awareness and understanding regarding cataracts and glaucoma among residents of Jazan, Saudi Arabia. With only a fraction of participants recognizing the symptoms and treatment options for these prevalent eye diseases, there is an urgent need for targeted public health initiatives. By enhancing educational efforts and promoting regular eye examinations, healthcare providers can significantly improve early detection and treatment outcomes, ultimately reducing the burden of visual impairment in the community. Addressing these gaps is essential for safeguarding eye health and improving overall quality of life for individuals at risk.

## Appendices

Survey questionnaire

1. Demographics

Age: ____

Gender: ( ) Male ( ) Female

Education: ( ) Primary ( ) Secondary ( ) Tertiary ( ) Other: ____

Occupation: ____

Family history of eye disease: ( ) Yes ( ) No

2. Cataracts Knowledge

Heard of cataracts? ( ) Yes ( ) No

Symptoms (select all):

Blurred vision ( ) Light sensitivity ( ) Night difficulty ( ) Halos ( ) Other: ____

Causes (select all):

Aging ( ) Eye injury ( ) Other: ____

Can cataracts be treated? ( ) Yes ( ) No

Treatment options (select all):

Surgery ( ) Medication ( ) Lifestyle changes ( ) Other: ____

3. Glaucoma Knowledge

Heard of glaucoma? ( ) Yes ( ) No

Symptoms (select all):

Peripheral vision loss ( ) Eye pain ( ) Blurred vision ( ) Headaches ( ) Other: ____

Causes (select all):

High eye pressure ( ) Family history ( ) Other: ____

Can glaucoma be treated? ( ) Yes ( ) No

Treatment options (select all):

Surgery ( ) Medication ( ) Other: ____

4. Eye Health

Frequency of eye exams:

Yearly ( ) Every 2-3 years ( ) Rarely ( ) Never

Importance of regular eye exams: ( ) Yes ( ) No

Main source of eye health info:

Doctors ( ) Internet ( ) Family/Friends ( ) Other: ____

## References

[1] Parrey MU, Abdul-Latif MM, Alruwaili SM, Alshammari KH, Alsayer RI, Alanazi NK, Abd El Mawgod MM (2024). Public awareness of common age-related eye diseases in northern Saudi Arabia. Cureus.

[2] Almesned RA, Jahan S (2024). Awareness about fall risk and measures of fall prevention among older adults in Buraidah, Saudi Arabia. Cureus.

[3] Alanazi AM, Abo El-Fetoh NM, Alotaibi HK (2017). Survey of awareness of diabetes mellitus among the Arar population, Northern Border Region of Saudi Arabia. Electron Physician.

[4] Morya RE, Alamoudi A, Ghaddaf AA, Taher NO, Almansour A, Alnahdi WA, Alghamdi S (2023). Public awareness about glaucoma, cataract, and diabetic retinopathy in Saudi Arabia: a systematic review and meta-analysis. Int Ophthalmol.

[5] Hajar S, Al Hazmi A, Wasli M, Mousa A, Rabiu M (2015). Prevalence and causes of blindness and diabetic retinopathy in Southern Saudi Arabia. Saudi Med J.

[6] Alharbi AM, Alhazmi AM (2020). Prevalence, risk factors, and patient awareness of diabetic retinopathy in Saudi Arabia: a review of the literature. Cureus.

[7] Du K, Guan H, Zhang Y, Ding Y, Wang D (2022). Knowledge of cataracts and eye care utilization among adults aged 50 and above in rural Western China. Front Public Health.

[8] Misra V, Vashist P, Singh SS, Malhotra S, Gupta V, Dwivedi SN, Gupta SK (2017). Awareness and eye health-seeking practices for cataract among urban slum population of Delhi: the North India eye disease awareness study. Indian J Ophthalmol.

[9] Dinesh Eshwar M, Jabeen A, Jalily QA, Begum GS (2022). Knowledge, awareness, and perception of common eye diseases and eye donation among people seeking healthcare in a tertiary hospital in Telangana, South India. Cureus.

[10] Albaqami FM, Saud Aljuaid A, Khalid Alrabie W, Abdulrahim Alotaibi M, Albaqami MM, Sultan Alharthi F, Alghamdi A (2023). Knowledge and awareness of glaucoma among people living in Taif City in the Western Region of Saudi Arabia. Cureus.

[11] Shrestha GS, Sigdel R, Shrestha JB, Sharma AK, Shrestha R, Mishra SK, Joshi SN (2018). Awareness of eye health and diseases among the population of the hilly region of Nepal. J Ophthalmic Vis Res.

[12] du Toit R, Ramke J, Naduvilath T, Brian G (2006). Awareness and use of eye care services in Fiji. Ophthalmic Epidemiol.

[13] Dandona R, Dandona L, John RK, McCarty CA, Rao GN (2001). Awareness of eye diseases in an urban population in southern India. Bull World Health Organ.

[14] Kamińska A, Pinkas J, Wrześniewska-Wal I, Ostrowski J, Jankowski M (2023). Awareness of common eye diseases and their risk factors—a nationwide cross-sectional survey among adults in Poland. Int J Environ Res Public Health.

[15] Ayanniyi AA, Olatunji FO, Adeboye A, Ayanniyi RO (2010). Awareness and knowledge of eye care providers among government workers in Ilorin, Nigeria. Niger Postgrad Med J.

